# Clinical Applications of Indocyanine Green Fluorescence Imaging in Vascular Malformations: A Systematic Review

**DOI:** 10.3390/jcm15051834

**Published:** 2026-02-27

**Authors:** Carlos Delgado-Miguel, Javier Arredondo-Montero, Julio César Moreno-Alfonso, Marta Rodríguez Ruiz, Isabella Garavis Montagut, Paloma Triana Junco, Miriam Miguel-Ferrero, Mercedes Díaz, Francisco Hernández-Oliveros, Juan Carlos López-Gutiérrez

**Affiliations:** 1 Pediatric Surgery Department, Fundación Jiménez Díaz University Hospital, 28040 Madrid, Spain; 2 Institute for Health Research IdiPAZ, La Paz University Hospital, 28046 Madrid, Spain; 3 Pediatric Surgery Department, Complejo Asistencial Universitario de León, 24071 León, Spain; 4 Pediatric Surgery Department, Navarra University Hospital, 31008 Pamplona, Spain; 5 Faculty of Medicine, El Bosque University, Bogotá 110121, Colombia; 6 Pediatric Surgery Department, La Paz University Hospital, 28046 Madrid, Spain

**Keywords:** indocyanine green, near-infrared fluorescence, vascular malformations, lymphatic vessels, capillary malformations, venous malformations, arteriovenous malformations

## Abstract

**Background/Objectives:** The use of near-infrared fluorescence (NIRF) imaging with indocyanine green (ICG) has gained increasing attention in the management of vascular malformations, offering real-time visualization of vascular and lymphatic structures that may improve surgical precision and outcomes. **Methods:** A systematic review was conducted in accordance with PRISMA guidelines, searching PubMed, Web of Science, CINAHL, and EMBASE databases for studies evaluating the intraoperative use of ICG in vascular malformations, which was prospectively registered in PROSPERO (CRD420251131951). Two independent reviewers screened all records based on predefined eligibility criteria. Extracted data included study design, patient characteristics, ICG administration protocols, clinical applications, and perioperative outcomes. **Results:** A total of 33 studies comprising 433 patients treated between 2014 and 2025 were included for qualitative synthesis. Nineteen (57.6%) were case reports, seven (21.2%) retrospective descriptive studies, two (6.1%) retrospective comparative studies, three (9.1%) prospective comparative trials, and two (6.1%) prospective descriptive studies. Clinical indications for ICG included capillary and venous malformations (5 studies), arteriovenous malformations (9 studies), and lymphatic malformations (19 studies). Quality assessment with the MINORS tool showed that most studies scored < 17, while only seven reached 18–24, reflecting higher methodological quality. **Conclusions:** Intraoperative ICG fluorescence imaging represents a promising adjunct in the treatment of vascular malformations, providing real-time visualization that may facilitate lesion delineation, guide resection, and support minimally invasive techniques such as lymphaticovenous anastomosis. However, current evidence is largely descriptive, with very limited comparative outcome data, and high-quality studies are needed to determine whether these technical advantages translate into improved long-term clinical outcomes.

## 1. Introduction

In recent years, the use of indocyanine green (ICG) fluorescence imaging has expanded across multiple medical disciplines due to its ability to provide real-time visualization of vascular structures and tissue perfusion [1,2,3]. In plastic and reconstructive surgery, ICG has been increasingly applied for flap perfusion assessment, sentinel lymph node mapping, and evaluation of microvascular anastomoses, demonstrating both safety and efficacy [4,5]. After intravenous injection, near-infrared fluorescence (NIRF) imaging allows real-time visualization of blood flow, identifying poorly perfused areas at risk of ischemia or necrosis [5]. This supports intraoperative decision-making and reduces complications like flap failure or delayed healing. ICG angiography is also used for lymphatic mapping to guide lymphaticovenous anastomosis in lymphedema, providing detailed perfusion maps that enhance reconstructive outcomes [6].

Despite this growing body of evidence, its application in the management of vascular malformations—including capillary, venous, arteriovenous, and lymphatic subtypes—remains less well characterized [7]. Vascular malformations present unique surgical and therapeutic challenges, often requiring precise delineation of abnormal vessels to optimize treatment outcomes while minimizing complications such as bleeding, ischemia, or recurrence [8]. Current interventions—including surgical excision or sclerotherapy—may benefit from accurate intraoperative visualization of lesion boundaries and feeding vessels. ICG fluorescence imaging offers a noninvasive, real-time method to achieve this goal [9,10].

To date, no systematic review has synthesized the clinical evidence regarding the use of ICG in this context. Given the heterogeneity of these lesions and the potential of ICG to improve intraoperative visualization, a comprehensive evaluation of the current literature is needed. This systematic review aims to fill this gap, providing a detailed overview of the clinical applications, dosage, administration routes, and outcomes associated with ICG fluorescence imaging in the management of vascular malformations in order to synthesize current evidence. Due to the predominance of descriptive and non-comparative studies in this field, this review is intended as a descriptive systematic review rather than a comparative effectiveness analysis.

## 2. Materials and Methods

### 2.1. Search Strategy

This systematic review was carried out following the guidelines established by the Preferred Reporting Items for Systematic Reviews and Meta-Analyses (Supplementary Material PRISMA) [11,12]. The research question was formulated using the PICO framework: “In patients with vascular malformations, does intraoperative ICG fluorescence imaging, compared to standard imaging techniques, improve surgical outcomes”? The PICO framework was adapted to the predominantly descriptive nature of the available evidence, focusing on patients—children or adults—with vascular malformations (capillary, venous, arteriovenous, or lymphatic) classified according to the ISSVA system (population), and evaluating the use of intraoperative or periprocedural ICG near-infrared fluorescence imaging (intervention). When present, the comparator consisted of standard imaging modalities or conventional management without ICG (comparator), although most studies did not include an explicit comparison group. The outcomes encompassed the reported clinical applications of the technique—such as diagnostic mapping, intraoperative guidance, and flow assessment—along with its technical feasibility, safety profile, and any perioperative or follow-up results, including complications, recurrence, or the need for re-intervention. This review was designed to characterize clinical use and technical applications of ICG fluorescence imaging rather than to formally compare effectiveness outcomes across interventions.

A comprehensive literature search was performed in four major electronic databases (PubMed, EMBASE, Web of Science, and CINAHL) on 23 September 2025. Because the search extended into late 2025, some recently published studies may still have limited dissemination or independent replication; however, they were included when available as full-text peer-reviewed articles within the searched databases. The search strategy combined controlled vocabulary (MeSH/Emtree terms) and free-text keywords related to indocyanine green and vascular malformations (capillary, venous, arteriovenous, and lymphatic). No publication date restrictions were applied. The gray literature sources (e.g., conference abstracts, theses, preprints, or registries) were excluded by design. The complete search strategies for each database are available in Supplementary Material S1.

The primary objective was to identify the clinical roles of ICG fluorescence imaging in the diagnosis, surgical planning, and intraoperative management of vascular malformations. Secondary objectives included evaluating its utility in assessing vascular flow patterns, defining lesion boundaries, determining optimal timing and dosage, and reporting safety data such as adverse reactions. This review was prospectively registered in the PROSPERO database under the ID CRD420251131951.

### 2.2. Eligibility Criteria

Studies were considered eligible if they involved human subjects (pediatric or adult populations) and reported the clinical use of ICG fluorescence imaging in the diagnosis, treatment, or follow-up of vascular malformations, including capillary, venous, arteriovenous, and lymphatic subtypes. Eligible publications were required to be full-text, peer-reviewed original articles providing clinical data on outcomes related to ICG use. Only studies written in English were included.

Exclusion criteria comprised editorials, opinion pieces, narrative reviews without original data, conference abstracts, theses, preprints, animal studies, basic experimental research, and any form of the gray literature. Duplicates were removed prior to screening. Initial selection was based on titles and abstracts, followed by a full-text review to confirm eligibility. Reference lists of included studies were manually searched to identify additional relevant articles (backward snowballing). In the present systematic review, primary lymphedema was not included, as it is not classified as a lymphatic malformation according to the International Society for the Study of Vascular Anomalies (ISSVA, 2018; updated 2025) [13]. While primary lymphedema represents a developmental disorder of the lymphatic system, its pathophysiology differs fundamentally from lymphatic malformations. ISSVA categorizes primary lymphedema under “complex lymphatic anomalies” rather than malformations, grouping it alongside conditions such as generalized lymphatic anomaly, Gorham–Stout disease, and kaposiform lymphangiomatosis. In contrast, lymphatic malformations are structural anomalies of lymphatic vessels characterized by cystic dilatation (macro-, micro-, or mixed cystic types) with abnormal endothelial proliferation. Given these distinct biological and clinical entities, primary lymphedema was excluded to maintain the homogeneity of the review population and ensure the focus remained on true lymphatic malformations.

### 2.3. Data Collection and Synthesis

From each study, data on study design, population characteristics, sample size, patient age, type of vascular malformation, ICG dosage and administration method, surgical or diagnostic technique applied, complications, and clinical outcomes were extracted. Two independent reviewers (CDM and IGM) completed the data collection using a standardized Microsoft Excel™ (2007, Redmond, WA, USA) template. Reference lists of the included studies were manually checked to capture additional relevant articles. Discrepancies were resolved by consensus after discussion. Because of the expected heterogeneity in study designs and clinical endpoints, a meta-analysis was not feasible. Instead, a narrative synthesis was performed, summarizing the clinical applications of ICG fluorescence imaging across different types of vascular malformations, along with practical aspects of administration and safety. As this was a systematic review of published data, ethical approval was not required. Extracted variables also included, when reported, markers of lesion severity (e.g., size, depth, ISSVA subtype, or other grading systems), presence of infection or associated complications, procedural success (such as completeness of resection or response to sclerotherapy), and duration of follow-up. However, these data were frequently incomplete or absent, particularly in case reports and small series.

### 2.4. Quality Appraisal

Quality appraisal was independently performed by two reviewers, and discrepancies were resolved through discussion and consensus, with the involvement of a third reviewer when necessary. The methodological quality of the included non-randomized studies was assessed using the Methodological Index for Non-Randomized Studies (MINORS) tool [14]. This scoring system evaluates 12 methodological criteria for comparative studies, with a maximum achievable score of 24 points. Initial scoring was carried out by one reviewer and independently verified by a second. Disagreements were resolved by involving a third reviewer. Studies with a score of ≥17 were considered high quality, whereas those scoring < 17 were deemed low quality. The MINORS tool was selected because most included studies were non-randomized surgical observational studies, for which MINORS provides validated methodological assessment criteria specifically designed for surgical research. ROBINS-I was not used due to the predominantly descriptive nature and small sample sizes of many included studies. The methodological quality of included case reports was assessed using the Joanna Briggs Institute (JBI) Critical Appraisal Checklist for Case Reports. Each study was evaluated across eight domains addressing patient description, diagnostic assessment, intervention reporting, outcome documentation, adverse events, and clinical relevance. Items were rated as “yes”, “no”, “unclear”, or “not applicable”. Given the descriptive nature of case reports, the assessment focused on reporting quality and risk of information bias rather than internal validity.

### 2.5. Certainty of Evidence

Although a quantitative meta-analysis was not feasible, we used the Grading of Recommendations Assessment, Development and Evaluation (GRADE) approach to qualitatively rate the certainty of evidence within three major clinical domains: (i) capillary and venous malformations, (ii) arteriovenous malformations, and (iii) lymphatic malformations. For each domain, we considered study design, risk of bias, inconsistency, indirectness, and imprecision. Certainty was categorized as high, moderate, low, or very low.

## 3. Results

**Figure 1:**
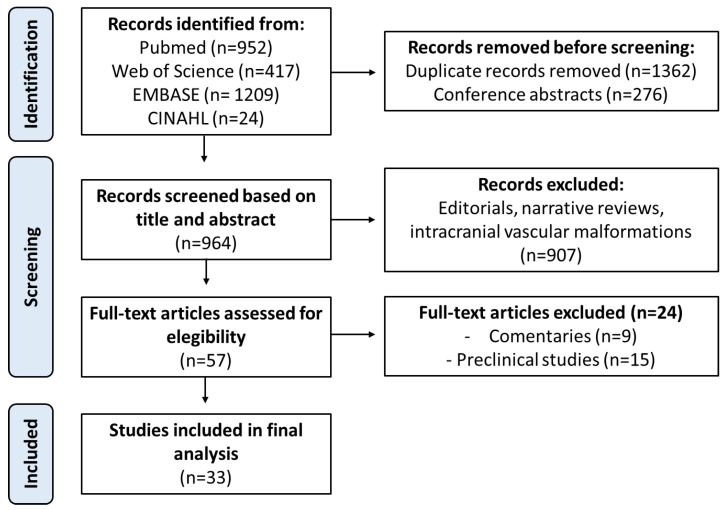
PRISMA flowchart.

A total of 2602 records were initially identified through database searches, of which 33 studies met the inclusion criteria and were incorporated into the narrative synthesis of this systematic review. In total, data from 433 patients with vascular malformations treated between 2014 and 2025 were analyzed. Of the included studies, 19 (57.6%) were case reports, 7 (21.2%) were retrospective descriptive studies, 2 (6.1%) were retrospective comparative studies, 3 (9.1%) were prospective single-center comparative trials, and 2 (6.1%) were prospective experimental descriptive studies. The PRISMA flowchart (Figure 1) illustrates the study selection process and reasons for exclusion.

**Figure 2:**
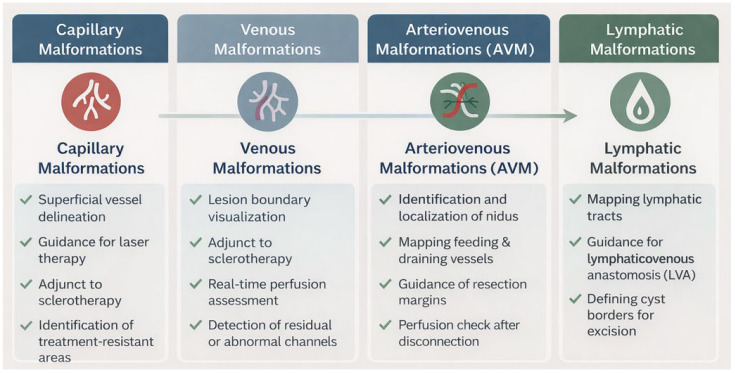
Summary of the main clinical applications of ICG imaging across different vascular malformation subtypes.

The main clinical applications of ICG in the management of vascular malformations—summarized in Table 1, Table 2 and Table 3—included capillary and venous malformations (5 studies), arteriovenous malformations (9 studies), and lymphatic malformations (19 studies). In contrast with capillary–venous and lymphatic malformations, for which comparative studies and multiple clinical case series are available, it is important to note that all the published literature on arteriovenous malformations consists exclusively of individual case reports. This narrow evidence base—lacking controlled comparisons, standardized outcome measures, and larger cohorts—limits the robustness of any conclusions that can be drawn and substantially lowers the certainty of the evidence, as the predominance of isolated cases hinders the ability to generalize findings or establish reliable clinical recommendations. Figure 2 provides a visual summary of the main clinical applications of ICG near-infrared fluorescence imaging across different vascular malformation subtypes. Considerable heterogeneity exists regarding ICG dosing, administration routes, and timing of imaging across studies. Reported doses ranged from 0.01 mg/mL to 4 mg/kg, with administration methods including intravenous, subcutaneous, intradermal, and intralesional injection. The timing of imaging relative to injection varied significantly depending on clinical indication. This lack of standardization represents a major barrier to guideline development and highlights the need for future protocol harmonization, particularly in pediatric populations where cumulative dosing and safety considerations are relevant.

Methodological quality, assessed with the MINORS tool, showed that more than half of the studies scored below 17, indicating low quality, while only seven studies achieved scores between 18 and 24, classifying them as higher quality (prospective or retrospective comparative studies). A detailed distribution of MINORS scores is available in Supplementary Material S2. Across the included case reports, the overall methodological quality assessed using the Joanna Briggs Institute Critical Appraisal Checklist was moderate to high, with most studies providing clear descriptions of patient demographics, clinical presentation, diagnostic workup, and intraoperative ICG-guided techniques (Supplementary Material S3). The majority of reports adequately detailed the surgical or imaging interventions and reported favorable postoperative outcomes. However, common sources of potential bias were related to the incomplete or unclear reporting of adverse events, limited or absent long-term follow-up in some studies, and the inherent lack of comparator groups associated with single-case designs. Consequently, although the reporting quality was generally acceptable, the descriptive nature of the evidence and the predominance of uncontrolled case reports substantially limit external validity and the certainty of the conclusions.

Using the GRADE framework, the certainty of evidence was rated as low to very low across all domains (Supplementary Material S4). This reflects the predominance of case reports and small non-comparative series, methodological limitations identified in the risk-of-bias assessment, heterogeneity in interventions and outcomes, and imprecise effect estimates. Consequently, current data should be interpreted as hypothesis-generating, and any potential benefits of ICG-NIRF should be viewed as tentative signals rather than established effects.

In 2012, Klein et al. demonstrated that intravenous ICG (2 mg/kg) combined with diode laser therapy (100 J/cm^2^) effectively coagulated telangiectatic leg veins in 15 female patients [15]. Using an NIR fluorescence system with an 835 nm filter, ICG was visualized, and laser treatment was initiated 2 min post-injection due to ICG’s short plasma half-life. They observed that ICG combined with diode laser treatment was safe with no lasting side effects, showing dose-dependent vessel clearance; single pulses at 100–110 J/cm^2^ achieved good results, improving to excellent with double pulses, whereas diode laser (DL) alone or pulsed dye laser (PDL) yielded only poor to moderate clearance of telangiectatic leg veins. Shortly thereafter, Ishikawa et al. evaluated the use of NIR fluorescence imaging after ICG administration in duplex-guided sclerotherapy performed on 15 patients with venous malformations in the lower extremities, upper extremities, and face [16]. Spotty fluorescence was observed in 87% of procedures and linear patterns in 53%, while no signal was detected in 13% (two cases) with intramuscular lesions of the lower extremities. The effective observation depth was <1 cm with 0.01 mg/mL ICG. No ICG-related complications occurred, though one patient developed adjacent tissue ulceration. More recently, Melley et al. used intraoperative laser-assisted ICG fluorescence angiography to delineate the extent of a glomuvenous malformation in a 19-year-old female [17]. A second 3 mg dose of ICG revealed three small satellite lesions, which were marked and resected, and a third dose with laser angiography confirmed complete excision, achieving full removal with no recurrence.

The first use of ICG in capillary malformations was reported by Klein et al. [18], who conducted a prospective, randomized controlled trial in 31 patients with port-wine stains, comparing single-session treatment with Flashlamp-Pumped Dye Laser (FPDL, 585 nm, 6 J/cm^2^, 0.45 ms) versus ICG combined with diode laser (DL, 810 nm, 20–50 J/cm^2^, 10–25 ms; ICG dose 2 mg/kg). Investigators observed slightly better clearance and aesthetics with ICG+DL, though not statistically significant (*p* = 0.114 and 0.291), while patients rated these outcomes significantly higher (*p* = 0.003 and 0.006). Histological analysis, including hematoxylin and eosin staining and CD34 immunostaining, showed that ICG+DL effectively induced photocoagulation in medium and large vessels (>20 µm), while smaller vessels remained largely unaffected. In 2013, a new randomized study was conducted by the same German group in 15 patients, increasing the ICG dose to 4 mg/kg [19]. Most patients had therapy-refractory capillary malformations and had previously received multiple PDL treatments, raising high expectations for the new approach. Both patients and the blinded investigator rated ICG+DL therapy slightly better than FPDL, although the difference did not reach statistical significance.

ICG has been applied in arteriovenous malformations (AVMs) at various anatomical sites, including the extremities, gastrointestinal tract, and pulmonary system. Akbayrak et al. reported the use of intravenous ICG in a 22-year-old with a symptomatic left forearm AVM [22]. ICG allowed near-infrared visualization of the nidus, feeding arteries, and draining veins, guiding en bloc resection with minimal bleeding. Fluorescence imaging was repeated to confirm complete removal, with no complications or transfusions and total blood loss under 100 mL.

Kurata et al. used intraoperative ICG angiography to define the resection line of a duodenal AVM during exploratory laparotomy, which revealed an enlarged right gastric vein, and ICG clearly delineated the lesion that was completely resected as planned [26]. Ono et al. combined selective angiography with intraoperative injection of ICG into the superior mesenteric artery, which allowed immediate recognition of the affected region by its green coloration. ICG fluorescence clearly highlighted dilated marginal arteries and focal pooling areas, enabling a targeted enterectomy of the AVM area, avoiding wider intestinal resections [20]. Another similar jejunal AVM case was reported by Hirakawa et al. in 2019 [21]. During double-balloon enteroscopy, a pulsatile submucosal uplift with a small red patch was observed in the jejunum, raising suspicion for an AVM, and temporary clips were placed to control arterial inflow. Multiple-phase CT and selective angiography identified multifocal niduses supplied by the second jejunal arteries. They performed an exploratory laparoscopy with intraoperative ICG injection (2.5 mg/mL, 0.2 mL), which highlighted a 30 cm segment, allowing precise segmental resection with minimal margins. In 2020, Hyo et al. reported a similar ileal AVM case using laparoscopic ICG fluorescence to locate the lesion and an infrared camera system to guide the resection line [23]. The mesenteric artery and small intestine appeared green 40 s after ICG injection, fading by 210 s, clearly defining the resection boundary. In 2023, Wagner et al. were the first to demonstrate the simultaneous intraoperative use of hyperspectral imaging and ICG in a gastrointestinal tumor with arteriovenous malformation, where both techniques assessed tumor extent and guided resection margins; intraoperative imaging confirmed an AVM, while histopathology revealed an epithelioid, partially spindle cell gastrointestinal stromal tumor [27].

Shiraishi et al. first reported the intraoperative use of ICG during resection of AVMs in the colorectal area (inferior mesenteric artery region) [24]. Laparoscopic exploration revealed thickening of the intestinal wall and mesentery from the sigmoid–descending colon junction to the upper rectum, with vascular hyperplasia surrounding the lesion. Intravenous ICG fluorescence imaging was used to evaluate intestinal and mesenteric perfusion, which was reconfirmed after mesenteric dissection to ensure adequate blood flow before specimen removal and again at the intestinal anastomosis to confirm blood flow. Johansson et al. described a pelvic mass in which near-infrared ICG imaging revealed efferent vessels from the left ovarian vein and a large communication with the external iliac vein; vascular clips were applied, the AVM was dissected with bipolar coagulation, and a second ICG dose confirmed hemostasis and organ perfusion [25]. ICG fluorescence has been used in pulmonary arteriovenous malformations (PAVMs), as reported by Han et al., who described two cases using intravenous ICG with NIRF to achieve intraoperative visualization of the lesions during video-assisted thoracoscopic surgery within just 10 s, allowing rapid and precise abnormal and normal tissue differentiation and facilitating limited resection of the PAVM without the need for more extensive tissue removal [28].

The first lymphatic malformation (LM) study reported was by Shibasaki et al. in 2014, who evaluated ICG lymphography for diagnosing and assessing lymphatic dysfunction in patients with generalized lymphatic dysplasia (GLD) presenting with congenital lymphatic pleural effusion and ascites [30]. Two imaging patterns were observed: linear flow, indicative of normal lymphatic drainage, and dermal backflow, reflecting dysfunction. Severity was further graded into four categories: mild, moderate, severe dysplasia, and hypoplasia. Clinical outcomes correlated with ICG findings, as all patients with mild or moderate dysplasia survived, whereas half of those with severe dysplasia died. In this context, Mihara et al. demonstrated that both the pleural effusion and ascites originated from lymphatic fluid draining from the extremities in eight neonates with GLD [31]. Based on these findings, lymphaticovenous anastomosis (LVA) was performed in five patients under general anesthesia through incisions <1 cm. Outcomes were assessed by monitoring ascites drainage from thoracic or abdominal tubes. Following LVA, effusions resolved completely in two patients and decreased in one; minimal dermal backflow was associated with resolution, whereas moderate backflow correlated with partial improvement. In 2017, Kato et al. used ICG lymphography to study lymphatic flow in a periorbital microcystic LM in an 11-month-old, enabling precise identification of afferent collecting lymphatic vessels [32]. This guided a subsequent LVA to bypass these vessels to a superficial vein, reducing lymphatic inflow to the lesion. In 2019, the same group analyzed in vivo lymphatic flow around LMs in 20 patients using ICG lymphography, identifying four flow patterns: type 1, strong inflow; type 2, multiple small inflows; type 3, superficial flow over the lesion; and type 4, flow surrounding the malformation without direct connection [35]. Later that year, they applied intraoperative subcutaneous ICG lymphography in 19 pediatric patients with mixed or microcystic LMs [36]. Patients with strong inflow underwent afferent lymph vessel-to-venous anastomosis, while others received LVA on the cyst wall. Macrocystic LMs or lesions suitable for complete resection were excluded. Within this framework, Furuse et al. reported a cystic submandibular LM treated with LVA under local anesthesia. ICG lymphography showed no inflow or outflow, so an outflow bypass was created using the LMVA sidewall technique [38]. The patient was discharged the next day without complications, and a six-month volumetric analysis showed a 43.5% reduction in malformation size, with complete patient satisfaction.

ICG has also been used intraoperatively to assess lymphatic location and flow. Han et al. applied pretreatment ICG lymphography in 71 patients with cervicofacial macrocystic LMs to identify lymphatic inflows and guide ultrasound-guided iodine tincture cauterization combined with posttreatment negative pressure, achieving excellent resolution in 87.3% and good improvement in 9.9% of cases [44]. Temporary ICG-related pigmentation occurred at injection sites in all patients, resolving within one month, and no iodine or ICG allergies were reported. The same German group performed ICG lymphography to evaluate the location and number of lymphatic inflows in macro- or mixed-cystic LMs in 81 children, guiding inflow occlusion combined with bleomycin sclerotherapy [41]. Detected inflows were visualized from distal to proximal regions, and a skin incision was made at the drainage site. Lymphatic vessels were dissected, confirmed by ICG, and occluded using bipolar electrocoagulation. The fluorescence probe was then used to verify occlusion and rule out leaks. A drainage tube was placed for bleomycin administration, and negative-pressure aspiration was removed on postoperative days 5–7 when no lymph remained. Excellent clinical outcomes were achieved in 68 patients (84%), with significant improvement in lesion appearance. More recently, comparative studies have been conducted to evaluate the effectiveness of intraoperative ICG use. Han et al. assessed ICG lymphography-guided resection and sclerotherapy for early-stage lingual microcystic LMs in 42 children (19 with ICGL guidance and 23 without) [45]. The ICG group showed superior efficacy and required fewer subsequent treatments, though operative times were longer. No significant differences were observed in postoperative complications, hospital stay, or follow-up duration. The same group analyzed the impact of intracystic hemorrhage (ICH) as a prognostic factor in 83 children with macro- or mixed-cystic lymphatic malformations, diagnosed via preoperative imaging and intraoperative ICG lymphography [46]. A complete absence of afferent lymphatic vessels was observed in ICH cases, supporting their isolated nature and identifying ICH as an independent, favorable prognostic factor despite its association with aggressive progression and compressive symptoms.

ICG-NIR fluorescence lymphatic imaging has been used to assess lymphatic involvement in systemic disorders. Rasmussen et al. evaluated a patient with Klippel–Trenaunay syndrome, finding dilated knee lymphatic vessels with preserved contractility but a reduced number compared to the contralateral limb [33]. Kanesi et al. used ICG in a very low birthweight infant with tuberous sclerosis, identifying linear vessels and dermal backflow indicative of lymphatic dysplasia [37]. Liu et al. reported two pediatric Noonan syndrome cases with pubic and groin lymphedema, where ICG showed slow, partial lymphatic flow, and magnetic resonance lymphangiography suggested lymphatic hyperplasia and functional impairment [39]. Several clinical cases have demonstrated the utility of ICG lymphangiography during LM surgery to guide safe dissection. Sharma et al. used NIRF to excise a recurrent peroneal LM while preserving the superficial peroneal nerve [29]. Drobot et al. mapped lymphatic flow around an axillary macrocystic LM, allowing excision while preserving vessels [42]. Kubota et al. visualized a single feeding lymphatic in an axillary lymphangioma for targeted removal [40]. Shirota et al. employed near-infrared fluorescence for abdominal wall LM, confirming lesion boundaries and removing adjacent abnormal lymphatic tissue, achieving complete resection without recurrence [34]. Menon et al. used ultrasound-guided ICG injection into superficial inguinal lymph nodes to identify the thoracic duct prior to ligation in cases of secondary chylothorax due to pulmonary lymphangiectasia with partial thoracic duct agenesis [47]. Takada et al. employed ICG lymphography to detect postoperative lymphatic leakage following resection of a thoracic lymphatic malformation and to guide thoracic duct ligation [43].

## 4. Discussion

This systematic review synthesizes the current evidence on ICG fluorescence imaging in the management of vascular malformations, a topic still underrepresented in the scientific field. Studies published over the last ten years were included, capturing the steady evolution of fluorescence technology and its translation into clinical management. Importantly, over 40% of the selected articles appeared within the most recent five years, highlighting accelerated adoption of the technique and a growing recognition of its potential to guide surgical planning and intraoperative decision-making in vascular malformations.

### 4.1. Capillary and Venous Malformations

In capillary malformations, ICG fluorescence has been explored as a strategy to improve laser treatment outcomes, particularly for lesions that are resistant to conventional pulsed dye laser therapy [18,19]. ICG acts as a photosensitizer, enhancing the absorption of laser energy by blood vessels and facilitating more effective photocoagulation, including smaller capillaries. This approach may expand treatment options for challenging vascular lesions, potentially improving clearance while maintaining acceptable cosmetic and safety outcomes. Its initial use in human patients was motivated by distinctive observations from two consecutive animal experiments investigating laser effects on blood vessels [48,49]. In the initial study, smaller vessels (diameter < 29 µm) were relatively resistant to FPDL therapy [48]. In a follow-up experiment using the same animal model, ICG-assisted diode laser treatment achieved irreversible photocoagulation across vessels of all sizes, including capillaries [49]. These findings supported the clinical exploration of ICG+DL, particularly for lesions refractory to conventional FPDL therapy. The therapeutic human use of systemic ICG with laser therapy for vascular lesions, including capillary malformations and telangiectatic veins, was first reported in 2012 at 2 mg/kg, later increased to 4 mg/kg, yielding good cosmetic outcomes without serious adverse effects [15,18]. However, significant differences compared with FPDL were not observed, likely due to the rapid decline of ICG after injection, reducing photothermal conversion. Immediate post-injection treatment was applied, though efficacy may diminish over time [50]. NIR fluorescence imaging, though limited to superficial lesions (<1 cm), provides real-time guidance during sclerotherapy and enhances safety when combined with duplex sonography. ICG has also proven valuable in glomuvenous malformations, where laser-assisted fluorescence imaging enables precise intraoperative margin delineation, improving excision completeness and potentially reducing recurrence [17]. Overall, in capillary and venous malformations, ICG consistently provides an improved visualization of vascular architecture and may enhance treatment precision, particularly in laser-based therapies. However, its impact on long-term clinical outcomes remains insufficiently demonstrated.

### 4.2. AVM Malformations

Arteriovenous malformations most frequently affect extremities but can also involve the gastrointestinal tract and lungs, each presenting unique clinical challenges. In extremity AVMs, ICG fluorescence imaging has emerged as a valuable intraoperative tool, allowing surgeons to visualize the nidus, its margins, and adjacent tissues in real time [22]. This complements preoperative imaging such as magnetic resonance imaging or computed tomography (CT) angiography, which provides detailed anatomical information but lacks dynamic intraoperative feedback, and offers advantages over two-dimensional digital subtraction angiography, particularly in patients with impaired renal function [20]. Despite its benefits, ICG imaging remains limited by device availability and reduced efficacy in deep or thrombosed lesions [22]. In the gastrointestinal tract, AVMs consist of hypertrophic, aberrant vessels that may mimic arterial structures and interconnect with thin-walled venous channels, often appearing as hemorrhagic spots, erosions, or polypoid lesions [51]. Defining lesion extent remains challenging. While CT and angiography estimate vascular involvement, intraoperative colonoscopy and selective ICG angiography provide real-time guidance for precise localization [21,23]. This targeted approach allows maximal preservation of healthy tissue, while fluorescence intensity offers functional insight—weak signals indicate ischemia, whereas strong signals highlight active angiogenesis [24,27]. Moreover, ICG can confirm adequate perfusion after mesenteric dissection, enhancing both safety and surgical precision. Pulmonary AVMs, frequently associated with hereditary hemorrhagic telangiectasia, consist of abnormal direct connections between pulmonary arteries and veins, bypassing the capillary bed, and are often difficult to identify with conventional imaging [52]. Systemic ICG injection during thoracic surgery highlights these abnormal pathways, differentiating them from normal lung parenchyma based on relative filling times and enabling precise excision, including small or peripheral lesions that might otherwise be overlooked, thus minimizing recurrence, pneumothorax risk, and collateral vessel formation [28]. From a hemodynamic perspective, fluorescence intensity patterns may reflect functional blood flow dynamics, including shunting, venous capacitance, and regional perfusion differences. These characteristics parallel principles observed in coronary and peripheral perfusion imaging, suggesting that ICG may provide functional as well as anatomical information during AVM surgery. Across different organ systems, ICG fluorescence imaging integrates anatomical and functional assessment in real time. This supports intraoperative decision-making, may improve resection completeness, and helps preserve functional tissue. These characteristics highlight the potential of ICG as an important adjunct in the surgical management of complex vascular malformations, which could reduce the proportion of patients presenting with bleeding complications [53]. Across AVM cases, ICG most consistently contributed to intraoperative identification of feeding vessels and resection margins, occasionally modifying surgical strategy by enabling more targeted resections. However, its effectiveness is limited in deeply located or thrombosed lesions.

### 4.3. Lymphatic Malformations

In lymphatic malformations, ICG can be applied through two complementary strategies, each providing distinct clinical benefits. Direct intralesional injection highlights the malformation itself, clearly delineating its borders to guide complete excision when no connections to normal lymphatics exist [39,40]. In contrast, peripheral injections, such as those administered between the digits, map surrounding lymphatic flow, enabling the surgeon to preserve functional vessels and perform careful dissection, thereby minimizing postoperative complications, including lymphatic leakage [29,35,36]. This technique provides real-time visualization of lymphatic anatomy and function and is particularly advantageous in pediatric patients because it is radiation-free and repeatable [54]. By combining minimally invasive injection with near-infrared imaging, ICG lymphography identifies afferent and efferent vessels. This guides inflow occlusion or limited cyst wall resection, even in anatomically complex regions such as the periorbital area or tongue [55,56]. LMs result from abnormal embryologic development of lymphatic endothelial cells, leading to cystic dilation that most commonly affects lymphoid-rich regions such as the head, neck, and extremities [57]. Accurate identification of lesion margins is critical, as incomplete resections often lead to recurrence, and conventional therapies such as sclerotherapy or wide surgical excision carry high complication rates [58]. ICG lymphography overcomes these limitations by providing a real-time, functional map of the lymphatic network, allowing selective reconstruction, lymphaticovenous anastomosis, or targeted excision with minimal tissue disruption [35,36]. Importantly, the dynamic nature of ICG visualization allows assessment of lymphatic flow patterns, confirmation of functional drainage, and detection of residual microlesions, which together improve surgical outcomes and reduce recurrence. The technique also facilitates monitoring in neonates and infants without sedation, while enabling the detection of lymphatic reflux or impaired pumping that may otherwise go unnoticed [59]. Clinical evidence demonstrates that preoperative and intraoperative ICG lymphography enhances lesion delineation, reduces the number of interventions, improves the efficacy of sclerotherapy or surgical excision, and preserves functional lymphatic anatomy, ultimately decreasing recurrence and postoperative complications [45,46,60]. In lymphatic malformations, ICG demonstrated consistent value in mapping lymphatic flow patterns and guiding surgical or reconstructive interventions, particularly in complex or pediatric cases. Nevertheless, evidence supporting improvement in long-term patient-centered outcomes remains limited.

### 4.4. Limitations

Despite the promising applications of ICG fluorescence imaging in vascular malformations, several limitations must be acknowledged. Most included studies were small, often single-center, and lacked randomized controlled designs, limiting the generalizability of findings and precluding meta-analytic synthesis. A key limitation of the available evidence is the predominance of case reports and small, single-center series, which account for more than half of the included studies. Although these reports provide hypothesis-generating observations and demonstrate technical feasibility, they are prone to selection and publication bias and cannot support robust causal inferences. In contrast, only a minority of studies employed comparative or cohort designs with systematic outcome reporting. Throughout this review, we therefore distinguish between findings arising from higher-level observational or comparative studies and those derived from isolated case reports, which should be interpreted as exploratory signals rather than definitive evidence. This imbalance in study design substantially lowers the overall certainty of evidence and underscores the need for larger, prospective, and controlled investigations. It should be emphasized that only a small subset of studies included a true comparator arm, so our PICO question is primarily descriptive and focused on characterizing clinical use and reported outcomes rather than formally comparing ICG-NIRF with standard imaging.

Heterogeneity in patient populations, lesion types, dosing protocols, imaging systems, and outcome measures complicates direct comparison between studies, as they were highly heterogeneous with respect to vascular malformation subtype (capillary, venous, arteriovenous, and lymphatic), anatomical location, ICG dose (ranging from 0.01 mg/mL to 4 mg/kg), route of administration (intravenous, subcutaneous, intradermal, or intralesional), and imaging systems. To provide a more structured synthesis, the findings can be grouped into three broad domains. First, in terms of diagnostic contribution, ICG-NIRF mainly served to delineate lesion margins, visualize flow patterns, and identify afferent and efferent vessels, particularly in lymphatic and arteriovenous malformations. Second, as an intraoperative guidance tool, ICG was used to define resection lines, guide sclerotherapy or inflow occlusion, and assist minimally invasive procedures such as lymphaticovenous anastomosis. Third, regarding postoperative outcomes, only sparse and largely descriptive data were available, with isolated reports suggesting reduced recurrence or improved cosmetic results, but without robust comparative endpoints. Given this heterogeneity, the results should not be interpreted as uniformly applicable across all malformation types or clinical scenarios, and the specific context of each study must be taken into account.

Interpretation of the clinical utility of ICG is further hampered by substantial gaps in the reporting of key variables. Lesion severity, depth, presence of infection, recurrence rates, completeness of resection, and standardized follow-up duration were rarely described in a consistent manner. Only a minority of studies provided objective measures of procedural success or long-term outcomes. As a result, it is difficult to determine whether ICG-NIRF is preferentially used in more complex or severe cases or whether its use is associated with any meaningful reduction in recurrence or complications. Improved clinical reporting will be essential to clarify the true impact of ICG fluorescence imaging. In addition, the depth penetration of ICG is limited to superficial structures (<1 cm), reducing its effectiveness in deeply located or thrombosed lesions, particularly in extremity and visceral AVMs [22,51]. Additionally, the availability of near-infrared imaging devices remains restricted in many centers, potentially limiting widespread clinical adoption. While the technique is generally safe, long-term outcomes and comparative efficacy against standard interventions remain insufficiently explored, emphasizing the need for well-designed prospective, multicenter studies to validate current findings and optimize protocols for diverse lesion subtypes. Future research should prioritize the development of standardized ICG administration protocols, the establishment of multicenter registries, and the evaluation of outcome-driven clinical endpoints, including recurrence, functional outcomes, and quality-of-life measures. Additionally, cost-effectiveness, learning curve, and equipment availability should be considered when evaluating widespread clinical adoption.

## 5. Conclusions

This systematic review highlights the growing role of ICG fluorescence imaging as a versatile adjunct in the management of vascular malformations. Across capillary, venous, arteriovenous, and lymphatic lesions, ICG provides real-time, high-resolution visualization of anatomy and function, facilitating precise lesion delineation, intraoperative decision-making, and preservation of normal tissue. In lymphatic malformations, the ability to map afferent and efferent vessels enables targeted excision, inflow occlusion, or lymphaticovenous anastomosis, improving surgical outcomes and reducing recurrence and postoperative complications. Similarly, in AVMs of the extremities, gastrointestinal tract, and lungs, ICG enhances identification of abnormal vasculature and confirms perfusion, contributing to safer, more complete resections. Overall, ICG fluorescence imaging demonstrates considerable potential as a technical adjunct to improve intraoperative visualization and support individualized treatment planning in vascular malformations. Nevertheless, the available evidence does not yet allow firm conclusions regarding its impact on recurrence, complication rates, or functional outcomes. Future prospective, comparative studies with standardized endpoints are required to clarify whether these promising intraoperative advantages ultimately translate into clinically meaningful benefits.

## Figures and Tables

**Table 1:** Capillary and venous malformations. Summary of ICG applications in capillary and venous malformations.

References (Country, Year)	Indication for ICG NIRF Surgery	Study Design	No. Patients	Age (Range)	Timing of ICG Injection	ICG Dose (Route)
Klein et al. (Germany, 2012) [15]	Evaluation of the safety and efficacy of ICG-augmented diode laser therapy (808 nm) of telangiectatic leg veins.	Prospective comparative	15	44 yr (28–62)	2 min after IV injection	2 mg/kg (IV)
Ishikawa et al. (Japan, 2013) [16]	Evaluation of the safety and efficacy of ICG fluorescence imaging in the sclerotherapy of venous malformations.	Prospective descriptive	15	Mean 14.9 yr (3–64)	Intraoperatively	0.01 mg/mL (direct puncture)
Melley et al. (USA, 2025) [17]	Usage of laser-assisted ICG fluorescent dye angiography in glomuvenous malformations surgery.	Case report	1	33 yr	Intraoperatively	2.5 mg (IV)
Klein et al. (Germany, 2012) [18]	Evaluation of the feasibility of ICG + DL in PWS and to compare it with FPDL in CM.	Prospective comparative	31	Median 33 yr (14–66)	Intraoperatively	2 mg/kg (IV)
Klein et al. (Germany, 2013) [19]	Evaluation of the safety and efficacy of ICG during augmented diode laser therapy in CM.	Prospective comparative	15	Median 25 yr (18–60)	Intraoperatively	4 mg/kg (IV)

yr, years; CM, capillary malformation; IV, intravenous; PWS, Port-Wine Stain; DL, diode laser; FPDL, flashlamp pumped pulsed dye laser.

**Table 2:** Arteriovenous malformations. Summary of ICG applications in arteriovenous malformations.

References (Country, Year)	Indication for ICG NIRF Surgery	Study Design	No. Patients	Age (Range)	Timing of ICG Injection	ICG Dose (Route)
Ono et al. (Japan, 2016) [20]	Locating gastrointestinal AVM (jejunum)	Case report	1	95 yr	Intraoperatively	2 mL of + 0.5% solution (IV)
Hirakawa et al. (Japan, 2019) [21]	Determining surgical margins in small bowel AVM	Case report	1	62 yr	Intraoperatively	0.2 mL of a 2.5 mg/mL solution (IV)
Akbayrak et al. (Turkey, 2019) [22]	Assessing AVM during surgery and identifying the feeding arteries	Case report	1	22 yr	Intraoperatively	25 mg (IV)
Hyo et al. (Japan, 2020) [23]	Locating and demarcate resection line (ileum AVM)	Case report	1	48 yr	Intraoperatively	5 mg (IV)
Shiraishi et al. (Japan, 2022) [24]	Confirming intestinal and mesenteric blood flow in small bowel AVM	Case report	1	70 yr	NS	NS
Johansson et al. (Australia, 2022) [25]	Locating pelvic AVM under robotic surgery	Case report	1	22 yr	Intraoperatively	3 mL of a 2.5 mg/mL (IV)
Kurata et al. (Japan, 2022) [26]	ICG angiography in duodenal AVM	Case report	1	18 yr	Intraoperatively	12.5 mg (IV)
Wagner et al. (Germany, 2023) [27]	ICG angiography in a GIST tumor with AVM	Case report	1	74 yr	Intraoperatively	NS
Han et al. (China, 2023) [28]	Visualizing the margin of pulmonary AVM	Case series	2	52 yr; 72 yr	Intraoperatively	25 mg (IV)

yr, years; NS, non-specified; AVM, arteriovenous malformation; IV, intravenous.

**Table 3:** Lymphatic malformations. Applications of ICG in the management of lymphatic malformations.

References (Country, Year)	Indication for ICG NIRF Surgery	Study Design	No. Patients	Age (Range)	Timing of ICG Injection	ICG Dose (Route)
Sharma et al. (UK, 2014) [29]	Visualizing and delineating the lymphatic and clearance during LM excision	Case report	1	33 yr	Intraoperatively	0.5 mL (ipsilateral first web space of the foot)
Shibasaki et al. (Japan, 2014) [30]	Evaluating lymphatic dysfunction and its association with clinical outcomes in GLD	Retrospective descriptive	10	Mean 67.6 days (1–275)	Intraoperatively	0.25 mg (SC)
Mihara et al. (Japan, 2015) [31]	Evaluating the lymphatic function in GLD with pleural effusion and ascites	Retrospective descriptive	8	0.5–7 mo	Every 3 h	0.1 mL (diagnogreen 0.05%) (intradermal)
Kato et al. (Japan, 2017) [32]	Determining the lymphatic flow pattern in the periorbital LM	Case report	1	11 mo	Intraoperatively	0.25 mg/mL; 12 injections of 0.02 mL (SC)
Rasmussen et al. (USA, 2017) [33]	Assessing the lymphatics in Klippel–Trenaunay Syndrome	Case report	1	32 yr	Intraoperatively	300 mcg (intradermal)
Shirota et al. (Japan, 2017) [34]	Visualizing the LM extension in the abdominal wall and confirm complete resection	Case report	1	15 yr	20 h before surgery	0.125 mg SC
Kato et al. (Japan, 2019) [35]	Evaluating and classify LM according to the observed patterns of in vivo lymph flow in LM during lymphography	Retrospective descriptive	20	Mean 4.5 yr (11 mo–10 yr)	Intraoperatively	0.25 mg/mL; 0.05 mL each in multiple spots (SC)
Kato et al. (Japan, 2019) [36]	Performing ICG fluorescence lymphangiography in microcystic or mixed-type LM	Retrospective descriptive	19	Mean 5.3 mo (0–11)	Intraoperatively	0.05 mL of 2.5 mg/mL (SC)
Kaneshi et al. (Japan, 2020) [37]	Evaluating lymphatic function in tuberous sclerosis complex	Case report	1	248 days	Intraoperatively	NS
Furuse et al. (Japan, 2020) [38]	Preoperatively assessing for performing lymphatic malformation venous anastomosis in LM	Case report	1	35 yr	Preoperatively	0.02 mL of 0.25 mg/mL (SC)
Liu et al. (China, 2020) [39]	Performing lymphangyography during surgery to make a safe dissection in LM	Case report	2	5 yr; 5 yr	Intraoperatively	NS
Kubota et al. (Japan, 2020) [40]	Performing lymphangyography during surgery to make a safe dissection in LM	Case report	1	35 yr	Intraoperatively	1 mL of 0.5 mg/mL (SC in para-areolar area)
Han et al. (China, 2021) [41]	Detecting afferent lymph vessels and rule out lymphatic leaks and inflow visualization in macro and mixed cystic LM	Prospective descriptive	81	6 mo–8 yr	Intraoperatively	0.05–0.1 mL in multiple sites of 2.5 mg/mL (intradermal and SC)
Drobot et al. (Israel, 2021) [42]	Performing lymphangyography during surgery to make a safe dissection in LM	Case report	1	14 yr	Intraoperatively	0.3–0.4 mL of 2.5 mg/mL (SC)
Takada et al. (Japan, 2023) [43]	Checking the presence of lymphatic leak after LM resection	Retrospective descriptive	1	6 yr	Preoperatively (no time specified)	0.1 mg/kg (SC)
Han et al. (China, 2022) [44]	Confirming lymph inflow within cervicofacial cystic LM	Retrospective descriptive	71	3 mo–7 yr	Intraoperatively	0.05–0.1 mL in multiple spots of 2.5 mg/mL (SC)
Han et al. (China, 2025) [45]	Evaluating the effectiveness and accuracy of ICG lymphography in lingual microcystic LM	Retrospective comparative	42 (19 ICG, 23 non-ICG)	IGC group: 4.6 ± 3.8 yr; non-ICG: 3.6 ± 2.6 yr	Intraoperatively	0.5 mg/kg (submucosal)
Han et al. (China, 2025) [46]	Examining the impact of intracystic hemorrhage on therapeutic outcomes in macro or mixed cystic LM	Retrospective comparative	83 (36 ICH group; 47 non-ICH)	NS	10 min before surgery	0.5 mg (intradermal)
Menon et al. (India, 2025) [47]	Identification of the thoracic duct before ligation in chylothorax secondary to pulmonary lymphangiectasia with partial thoracic duct agenesis	Retrospective descriptive	1	1.5 yr	Intraoperatively	2.5 mg (US-guided injection in superficial inguinal lymph nodes)

yr, years; mo, months; NS, non-specified; LM, lymphatic malformation; SC, subcutaneous; GLD, generalized lymphatic dysplasia; US, ultrasound; ICH, intracystic hemorrhage.

## Data Availability

Data collection form templates, data extracted from included studies, and any other materials used in the review can be requested from the corresponding author of the study.

## Supplementary Materials

The following supporting information can be downloaded at https://www.mdpi.com/article/10.3390/jcm15051834/s1. Supplementary Material S1: Search strategy for the systematic review. Supplementary Materials S2: MINORS scores of the included studies. Supplementary Materials S3: JBI scores of the included case reports. Supplementary Materials S4: GRADE assessment of the certainty of evidence for each clinical domain; PRISMA 2020 Checklist.
