# Supplementary material for: Clinical Applications of Indocyanine Green Fluorescence Imaging in Vascular Malformations: A Systematic Review
Source: J Clin Med. 2026 Feb 27;15(5):1834. doi: 10.3390/jcm15051834 (PMC12985441; doi:10.3390/jcm15051834)
Supplement: Supplementary file 1 [file jcm-15-01834-s001.zip › Supplementary material-S1-S4.pdf]

Supplementary Material S1. Search strategy for the systematic review.

Database	Platform	Date searched	Search strategy	Records retrieved
**PubMed**	NLM	23/08/2025	("Indocyanine Green"[Mesh] OR "Indocyanine Green"[tiab] OR "ICG"[tiab] OR "Fluorescence-Guided Surgery"[Mesh] OR "fluorescence-guided"[tiab]) AND ("Vascular Malformations"[Mesh] OR "vascular malformation*"[tiab] OR "Lymphatic Abnormalities"[Mesh] OR "lymphatic malformation*"[tiab] OR "Capillary Malformations"[Mesh] OR "capillary malformation*"[tiab] OR "Arteriovenous Malformations"[Mesh] OR "arteriovenous malformation*"[tiab])	952
**EMBASE**	Elsevier	23/08/2025	('indocyanine green'/exp OR 'indocyanine green':ti,ab OR 'ICG':ti,ab OR 'fluorescence guided surgery'/exp OR 'fluorescence-guided':ti,ab) AND ('vascular malformation'/exp OR 'vascular malformation*':ti,ab OR 'lymphatic malformation'/exp OR 'lymphatic malformation*':ti,ab OR 'capillary malformation'/exp OR 'capillary malformation*':ti,ab OR 'arteriovenous malformation'/exp OR 'arteriovenous malformation*':ti,ab)	1209
**Web of Science Core Collection**	Clarivate	23/08/2025	TS=("indocyanine green" OR "ICG" OR "fluorescence-guided") AND TS=("vascular malformation*" OR "lymphatic malformation*" OR "capillary malformation*" OR "arteriovenous malformation*")	417
**CINAHL**	EBSCO	23/08/2025	(MH "Indocyanine Green" OR "indocyanine green" OR ICG OR "fluorescence-guided") AND (MH "Vascular Malformations" OR "vascular malformation*" OR MH "Lymphatic Abnormalities" OR "lymphatic malformation*" OR "capillary malformation*" OR "arteriovenous malformation*")	24

Supplementary Material S2. MINORS scores of the included studies.

Reference	Adequate statistical analyses	Baseline equivalence of groups	Clearly stated aim	Contemporary groups	Control group used	Endpoints appropriate to the aim of the study	Follow-up period appropriate	Inclusion of consecutive patients	Loss to follow-up less than 5%	Prospective calculation of the study size	Prospective data collection	Unbiased assessment of the study endpoint	Total Score
Klein et al (Germany, 2012)	2	2	2	2	2	2	2	2	2	2	2	2	24
Ishikawa et al (Japan, 2013)	2	0	2	0	0	2	2	2	2	2	2	2	18
Klein et al (Germany, 2012)	2	2	2	2	2	2	2	2	2	2	2	2	24
Klein et al (Germany, 2013)	2	2	2	2	2	2	2	2	2	2	2	2	24
Ono et al. (Japan, 2016)	2	0	2	0	0	2	2	2	2	0	0	2	14
Hirakawa et al. (Japan, 2019)	2	0	2	0	0	2	2	2	2	0	0	2	14
Akbayrak et al. (Turkey, 2019)	2	0	2	0	0	2	2	2	2	0	0	2	14
Hyo et al. (Japan, 2020)	2	0	2	0	0	2	2	2	2	0	0	2	14
Shiraishi et al. (Japan, 2022)	2	0	2	0	0	2	2	2	2	0	0	2	14
Johansson et al. (Australia, 2022)	2	0	2	0	0	2	2	2	2	0	0	2	14

Kurata et al. (Japan, 2022)	2	0	2	0	0	2	2	2	2	0	0	2	14
Wagner et al. (Germany, 2023)	2	0	2	0	0	2	2	2	2	0	0	2	14
Han et al. (China, 2023)	2	0	2	0	0	2	2	2	2	0	0	2	14
Sharma et al. (UK, 2014)	2	0	2	0	0	2	2	2	2	0	0	2	14
Shibasaki et al. (Japan, 2014)	2	0	2	0	0	2	2	2	2	0	0	2	14
Mihara et al. (Japan, 2015)	2	0	2	0	0	2	2	2	2	0	0	2	14
Kato et al. (Japan, 2017)	2	0	2	0	0	2	2	2	2	0	0	2	14
Rasmussen et al (USA, 2017)	2	0	2	0	0	2	2	2	2	0	0	2	14
Shirota et al. (Japan, 2017)	2	0	2	0	0	2	2	2	2	0	0	2	14
Kato et al. (Japan, 2019)	2	0	2	0	0	2	2	2	2	0	0	2	14
Kato et al. (Japan, 2019)	2	0	2	0	0	2	2	2	2	0	0	2	14
Kaneshi et al. (Japan, 2020)	2	0	2	0	0	2	2	2	2	0	0	2	14
Furuse et al. (Japan, 2020)	2	0	2	0	0	2	2	2	2	0	0	2	14
Liu et al. (China, 2020)	2	0	2	0	0	2	2	2	2	0	0	2	14
Kubota et al. (Japan, 2020)	2	0	2	0	0	2	2	2	2	0	0	2	14
Han et al. (China, 2021)	2	0	2	0	0	2	2	2	2	2	2	2	18

Drobot et al. (Israel, 2021)	2	0	2	0	0	2	2	2	2	0	0	2	14
Takada et al. (Japan, 2022)	2	0	2	0	0	2	2	2	2	0	0	2	14
Han et al. (China, 2022)	2	0	2	0	0	2	2	2	2	0	0	2	14
Han et al. (China, 2025)	2	2	2	2	2	2	2	2	2	0	0	2	20
Han et al. (China, 2025)	2	2	2	2	2	2	2	2	2	0	0	2	20
Melley et al. (USA, 2025)	2	0	2	0	0	2	2	2	2	0	0	2	14
Menon et al. (India, 2025)	2	0	2	0	0	2	2	2	2	0	0	2	14

Supplementary Material S3. JBI scores of the included case reports.

Reference	Study Design	Were the patient's demographic characteristics clearly described?	Was the patient's history clearly described and presented as a timeline?	Was the current clinical condition of the patient on presentation clearly described?	Were diagnostic tests or assessment methods and the results clearly described?	Was the intervention(s) or treatment procedure(s) clearly described?	Was the post-intervention clinical condition clearly described?	Were adverse events or unanticipated events identified and described?	Does the case report provide clear take-away lessons or clinical implications?	Score
Ono et al. (Japan, 2016)	Case report	YES	YES	YES	YES	YES	YES	UNCLEAR	YES	Moderate-High
Hirakawa et al. (Japan, 2019)	Case report	YES	YES	YES	YES	YES	YES	UNCLEAR	YES	Moderate-High
Akbayrak et al. (Turkey, 2019)	Case report	YES	YES	YES	YES	YES	YES	YES	YES	High
Hyo et al. (Japan, 2020)	Case report	YES	YES	YES	YES	YES	YES	YES	YES	High
Shiraishi et al. (Japan, 2022)	Case report	YES	YES	YES	YES	YES	YES	YES	YES	High
Johansson et al. (Australia, 2022)	Case report	YES	YES	YES	YES	YES	YES	YES	YES	High
Kurata et al. (Japan, 2022)	Case report	YES	YES	YES	YES	YES	YES	YES	YES	High
Wagner et al. (Germany, 2023)	Case report	YES	YES	YES	YES	YES	YES	YES	YES	High

Han et al. (China, 2023)	Case series	YES	YES	YES	YES	YES	YES	YES	YES	High
Sharma et al. (UK, 2014)	Case report	YES	YES	YES	UNCLEAR	YES	UNCLEAR	UNCLEAR	YES	Moderate
Kato et al. (Japan, 2017)	Case report	YES	YES	YES	YES	YES	YES	YES	YES	High
Rasmussen et al (USA, 2017)	Case report	YES	YES	YES	YES	Not applicable	YES	YES	YES	Moderate
Shirota et al. (Japan, 2017)	Case report	YES	YES	YES	YES	YES	YES	YES	YES	High
Kaneshi et al. (Japan, 2020)	Case report	YES	YES	YES	YES	Not applicable	YES	UNCLEAR	YES	Moderate-High
Furuse et al. (Japan, 2020)	Case report	YES	YES	YES	YES	YES	YES	YES	YES	High
Liu et al. (China, 2020)	Case report	YES	YES	YES	YES	YES	YES	YES	YES	High
Kubota et al. (Japan, 2020)	Case report	YES	YES	YES	YES	YES	YES	YES	YES	High
Drobot et al. (Israel, 2021)	Case report	YES	YES	YES	YES	YES	YES	YES	YES	High
Melley et al. (USA, 2025)	Case report	YES	YES	YES	YES	YES	YES	YES	UNCLEAR	Moderate-High

Supplementary Material S4. GRADE assessment of the certainty of evidence for each clinical domain

Clinical domain	Study designs included	Study limitations (risk of bias)	Inconsistency	Indirectness	Imprecision	Publication bias	Overall GRADE certainty	Conclusion
**Lymphatic malformations (LM)**	1 RCT, 3 prospective comparative studies, 6 retrospective series, 9 case reports	**Serious** (predominance of non-comparative designs; small samples)	**Serious** (high variability in technique, dosing, and endpoints)	Not serious	**Serious** (few patients; heterogeneous outcomes)	Likely	**Very low**	Evidence suggests potential usefulness for mapping and surgical guidance, but does not allow conclusions regarding clinical outcome improvement.
**Venous & capillary malformations (VM/CM)**	3 prospective comparative studies, 1 prospective	**Serious** (high risk of bias, small samples, non-	**Serious** (wide variability in laser parameters, dosing, protocols)	**Serious** (limited generalizability to deeper lesions)	**Serious**	Likely	**Low**	Evidence is insufficient to determine efficacy; ICG may aid visualization, but has not

Clinical domain	Study designs included	Study limitations (risk of bias)	Inconsistency	Indirectness	Imprecision	Publication bias	Overall GRADE certainty	Conclusion
	descriptive study, 1 case reports	comparative designs)						been shown to improve clinical outcomes.
**Arteriovenous malformations (AVM)**	No randomized trials; 1 retrospective series; 8 case reports	**Critical** (almost all are case reports without controls)	**Serious** (different anatomical sites, techniques, and objectives)	**Serious**	**Critical** (minimal sample sizes; absent standardized outcomes)	Likely	**Very low**	ICG appears promising for real-time identification of nidus and perfusion, but no evidence supports improvement in safety or outcomes.
**Overall evidence**	Majority observational; 57% are case reports	**Critical**	**Serious**	**Serious**	**Critical**	Highly likely	**Very low**	Overall certainty is **very low**; conclusions must be interpreted with extreme caution.
